# C-Sibelia: an easy-to-use and highly accurate tool for bacterial genome comparison

**DOI:** 10.12688/f1000research.2-258.v1

**Published:** 2013-11-25

**Authors:** Ilya Minkin, Hoa Pham, Ekaterina Starostina, Nikolay Vyahhi, Son Pham

**Affiliations:** 1 Bioinformatics Institute, St. Petersburg, Russian Federation; 2 GNT Incorporation, HoChiMinh City, Vietnam; 3 Computer Science and Engineering, University of California San Diego, La Jolla, 92092, USA

## Abstract

We present C-Sibelia, a highly accurate and easy-to-use software tool for comparing two closely related bacterial genomes, which can be presented as either finished sequences or fragmented assemblies. C-Sibelia takes as input two FASTA files and produces: (1) a VCF file containing all identified single nucleotide variations and indels; (2) an XMFA file containing alignment information. The software also produces Circos diagrams visualizing high level genomic architecture for rearrangement analyses. C-Sibelia is a part of the Sibelia comparative genomics suite, which is freely available under the GNU GPL v.2 license at http://sourceforge.net/projects/sibelia-bio. C-Sibelia is compatible with Unix-like operating systems. A web-based version of the software is available at http://etool.me/software/csibelia.

## Introduction

The development of inexpensive genome sequencing technologies and efficient assembly methods has revolutionized the study of bacterial genomes, which are being sequenced and assembled on a daily basis. When an assembly is available, the most common first task is to compare it against a reference genome (or another assembly, if no such genome is available) in order to find genetic differences between the newly assembled and reference genomes. This analysis is critical to understand genetic factors that determine certain phenotypes of the isolates.

We present Comparative Sibelia software (C-Sibelia) for the comparison of two bacterial genomes in the form of complete sequences or draft assemblies. C-Sibelia is able to compare genomes in the presence of rearrangements and duplications. C-Sibelia takes as input two FASTA files (the assembly and reference files; if the reference genome is not available, it can be substituted by another draft assembly) and produces: (1) a VCF file containing all identified single nucleotide variations (SNVs) and indels; (2) annotation of these variants by SnpEff; (3) an XMFA ^1^ file containing alignment information. The web-based version also produces a circular diagram visualizing the rearrangement of synteny blocks in two genomes.

The performance of C-Sibelia in detecting SNVs and indels is comparable to MUMmer and outperforms Mauve in terms of the false-positive rate. C-Sibelia is a part of the Sibelia comparative genomics suite, which is freely available under the GNU GPL v.2 license at http://sourceforge.net/projects/sibelia-bio. Users are encouraged to use the web-based version of C-Sibelia at http://etool.me/software/csibelia.

## Methods

### From synteny blocks to alignment

The task of finding SNVs and indels connects closely to the problem of whole-genome alignment. Unlike aligning two short DNA segments, aligning two genomes is more challenging because of the presence of rearrangements and repetitive elements. C-Sibelia addresses this problem by first decomposing genomes into synteny blocks, using the *iterative de Bruijn graph algorithm* described in Minkin *et al.* ^2^. This step separates linear operations (indels, substitutions) from non-linear operations (rearrangements) and thus allows us to apply global alignment to multiple instances of each synteny block. C-Sibelia incorporates LAGAN ^3^, a global alignment tool, for aligning different instances of the same synteny block.

***From alignment to variant calling.*** C-Sibelia then finds differences between two genomes (indels, SNVs, rearrangements) by analyzing the resulting synteny and alignment blocks. Regions in one genome not covered by synteny blocks are treated as indels. SNVs and small indels that lie within the regions covered by synteny blocks are reported by analyzing the alignment information produced by LAGAN. Identified variants are annotated by using snpEff ^4^. The pipeline of C-Sibelia is described in the following pseudocode.

**Input**: An assembly and a reference genome (in FASTA format).

**Algorithm**:

- Decompose the sequences into synteny blocks using Sibelia.
- Align instances of synteny blocks using LAGAN.
- Analyze the synteny block decomposition and alignment information.

            – Find indels in non-syntenic regions.

            – Find small indels and SNVs in aligned regions (using the alignment information produced by LAGAN).

            – Annotate the identified variants using SnpEff.

            – Select contigs containing multiple synteny blocks (i.e., rearranged contigs).

**Output**:

- All SNVs and indel variants, in a VCF file.
- Annotation of these variants produced by SnpEff ^4^.
- A picture in Circos format ^5^ for rearranged contigs and the reference genome.

## Results

### A simulated dataset

To evaluate the variant calling feature, we benchmarked C-Sibelia against Mauve ^6^ and MUMmer ^7^ on a simulated dataset, designed as follows.

**Figure 1.**
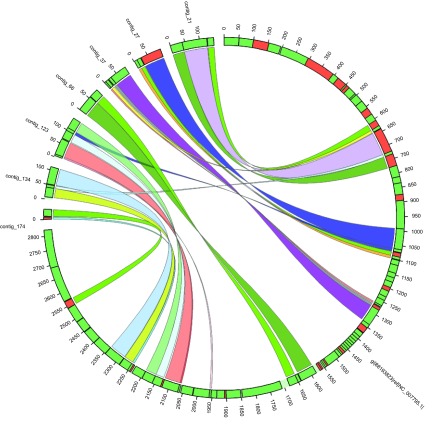
A picture in Circos format for assembly sequences and the reference genome. Only contigs with multiple synteny blocks rearranged differently in the genome are shown. Green and red bars depict the direction of synteny blocks on the positive and negative strands, respectively.

From the complete genome of *Staphylococcus aureus* ( *S. aureus*) NCTC 8325, we performed 10 deletions of random segments of size 2000 bp, and futher introduced 1000 SNVs in the resulting genome. We then generated five reversals and five translocations of random segments in the genome with size 10,000 bp each to evaluate the capability of these tools to perform an alignment in the presence of rearrangements. We obtained a *simulated assembly* of this newly *simulated genome* of 180 contigs; the distribution of contig length was similar to that of the RN4220 assembly reported in Dhanalakshmi *et al.* ^8^. We further used C-Sibelia, Mauve and MUMmer to find variants in this simulated assembly and the original reference genome (NCTC 8325). Table 1 and Table 2 demonstrate that the performance of C-Sibelia in detecting variants is comparable to MUMmer and improves upon Mauve in terms of the false-positive rate. Figure 1 shows the Circos diagram of the rearranged contigs and the reference genome. The scripts and commands used for this benchmark are available in the Supplementary material.

**Table 1.** SNV calling on simulated data.

Tool	True Positive	False Positive	False Negative
C-Sibelia	976	0	24
MUMmer	977	0	23
Mauve	991	78	9

**Table 2.** Indel calling on simulated data.

Tool	True Positive	False Positive	False Negative
C-Sibelia	9	0	1
MUMmer	9	0	1
Mauve	10	1	0

### A real dataset

The most common approach for comparing an assembly against a reference genome is to first align the assembly against the reference and then write in-house scripts to extract variants. C-Sibelia can achieve this task automatically and with high accuracy. We used C-Sibelia to reproduce the comparison of the *S. aureus* RN4220 assembly and the reference genome NCTC 8325, reported in Dhanalakshmi *et al.* ^8^ (the authors used MUMmer and in-house scripts for this comparison). Among 132 single nucleotide variants and four large deletions reported in Dhanalakshmi *et al.* ^8^, C-Sibelia confirmed 121 SNVs and all four large deletions. C-Sibelia also reported six additional variants, which are also confirmed by BLAST ^9^. The input data as well as the commands for generating these results are available in the Supplementary material.

### The Etool Web-Server

The online version of C-Sibelia is available at http://etool.me/software/csibelia. The web form takes as input two FASTA files (one for the assembly and the other for the reference). The web form’s parameters allow users to choose whether or not to annotate variants and display the Circos ^5^ picture for rearrangement analysis (see Figure 1). Results are delivered to registered users by a real time push notification mechanism ^10, 11^.

## Discussion

In this application note, we introduced C-Sibelia, a novel software for comparing two closely-related bacterial strains. Performance of C-Sibelia is comparable to MUMmer, and better than Mauve in terms of false positives rate. The web interface of C-Sibelia makes the task of comparing assemblies against a reference genome convenient for microbiologists, who do not want to go to the trouble of downloading and compiling the software. In the future, we plan to extend C-Sibelia to compare multiple genomes or draft assemblies as well as scale the software to larger genomes.

## Supplementary material

Supplemenary material can be found online at http://goo.gl/jtLsPl and is permanently available at doi: 10.5281/zenodo.7577.
