# Supplementary material for: C-Sibelia: an easy-to-use and highly accurate tool for bacterial genome comparison
Source: F1000Res. 2013 Nov 25;2:258. [Version 1] doi: 10.12688/f1000research.2-258.v1 (PMC4111117; doi:10.12688/f1000research.2-258.v1)
Supplement: Peer review history

# Peer review: Referee response for version 1

**DOI:** 10.5256/f1000research.2867.r4953

**Published:** 2014-07-17

**Authors:** Loren J Hauser

**Affiliations:** 1 Computational Biology and Bioinformatics Group, Biosciences Division, Oak Ridge National Laboratory, Oak Ridge, TN, USA

C-Sibelia was created to be a user friendly tool for pairwise genome comparison. The web tool is relatively easy to use for the non-bioinformatics trained and will therefore make these kinds of analysis easier for small groups to perform. This is a big need. I can see the possibility of additional analyses to be added which will enhance the site. However, the output page has some real deficiencies that need to be fixed prior to public use.

 

- The web site can’t recognize a fasta file unless named xxx.fasta. This is annoying since all genbank fasta files end in .fna and therefore will have to be renamed before use. This is an easy fix.
- I tested 2 bacterial genomes over 6 Mb and it took about 5 minutes for the results to come back. This is OK but could be a problem if the site eventually gets a lot of use.
- The output needs a lot more definitions or explanations: for example 1) the section titled “ *number of effects by impact*”, I have no idea what high, low, moderate or modifier means; 2) there are 10,853 modifiers but there are only 1037 listed in the section “ *changes by type*”; 3) in the section “ *number of effects by functional class”* there are 36 missense and 75 silent mutation listed, but there are 160 non-synonymous_coding and 136 synonymous_coding in the section “ *number of effect by type and region*”; 4) since these were bacterial genomes how are exons defined?
- There are additional inconsistencies and lack of definitions that I have not pointed out.

The best way to fix this is to get somebody who has never used the system and has not been trained in any way to test it in order to point out all of these inconsistencies and lack of definitions.

Some of the output was unavailable since in ran off the bottom of the allotted space. This will be a real problem when comparing draft genomes with more than 10 contigs. I did not get to see the circular map or the distribution of changes on the main chromosome because of this.

I did not try to download and use the executable, but if it as poorly described as the output it will be hard for novices to implement. Again, get a complete novice to test downloading and installing it to make sure it is easy to use.

I have read this submission. I believe that I have an appropriate level of expertise to confirm that it is of an acceptable scientific standard.

# Peer review: Referee response for version 1

**DOI:** 10.5256/f1000research.2867.r2566

**Published:** 2013-11-27

**Authors:** Jeffrey McLean

**Affiliations:** 1 Microbial and Environmental Genomics, J. Craig Venter Institute, San Diego, CA, USA

In general, the rationale behind the decision to develop a user friendly tool to compare finished and draft assemblies, to reference genomes, is highly justified. The accurate calling of single nucleotide variations and indels using the comprehensive SnpEff tool will allow a wide variety of users to make use of C-Sibelia. For non-informatic inclined users, the output formats and the optional Circos diagrams visualizing high level genomic architecture for rearrangement analyses, is an excellent addition. The framework for the software is also an interesting choice, making it accessible to social networking style discussions with other users.

On the technical side, the authors do demonstrate that the performance of C-Sibelia in detecting variants is comparable to MUMmer and improves upon Mauve in terms of the false-positive rate. These tools are currently highly used and the research community would benefit from a user friendly tool such as C-Sibelia. The approach to decompose the genomes into synteny blocks, using the iterative de Bruijn graph algorithm is novel *. *In order to validate this tool I have signed in to the web-based version (signing in increases the size of the files you can upload) and tested an assembly against a reference genome and obtained results comparable to MAUVE. An explanation of the common use of the VCF format would help readers further. Visualizations of the SNPs would also be a nice addition in the future. I look forward to further enhancements of this tool.

I have read this submission. I believe that I have an appropriate level of expertise to confirm that it is of an acceptable scientific standard.
